# 3′-*O*-Acetyl-2′-de­oxy­uridine

**DOI:** 10.1107/S160053681004938X

**Published:** 2010-12-04

**Authors:** Bogdan Doboszewski, Alexander Y. Nazarenko, Victor N. Nemykin

**Affiliations:** a Departamento de Química, Universidade Federal Rural de Pernambuco, 52171-900 Recife, PE, Brazil; b Chemistry Department, State University of New York, College at Buffalo, 1300 Elmwood Ave, Buffalo, NY 14222-1095, USA; c Department of Chemistry & Biochemistry, University of Minnesota Duluth, Duluth, Minnesota 55812-2496, USA

## Abstract

In the two independent but very similar mol­ecules of the title compound, C_11_H_14_N_2_O_6_, both nucleobase fragments are nearly planar (both within 0.01 Å) while the furan­ose rings exhibit ^2^ *E*-*endo* envelope conformations. In the crystal, the two 3′-*O*-acetyl-2′-de­oxy­uridine mol­ecules form a pseudosymmetric dimer of two bases connected *via* two nearly identical resonance-assisted N—H⋯O hydrogen bonds. The resulting pair is further connected with neighboring pairs *via* two similar O—H⋯O bonds involving the only hydroxyl group of the 2′-de­oxy­furan­ose fragment and the remaining carbonyl oxygen of the nucleobase. These inter­actions result in the formation of an infinite ‘double band’ along the *b* axis that can be considered as a self-assembled analogue of a polynucleotide mol­ecule with non-canonical Watson–Crick base pairs. The infinite chains of 3′-*O*-acetyl-2′-de­oxy­uridine pairs are additionally held together by C—H⋯O inter­actions involving C atoms of the uracyl base and O atoms of carbonyl groups. Only weak C—H⋯O contacts exist between neighboring chains.

## Related literature

For syntheses of this and similar compounds, see: Smrt & Sorm (1960 ▶); Cabral *et al.* (2008 ▶). For related structures of uridines, see: de Graaff *et al.* (1977 ▶); Green *et al.* (1975 ▶); Low & Wilson (1984 ▶); Luo *et al.* (2007 ▶); Marck *et al.* (1982 ▶); Rahman & Wilson (1972 ▶); Suck *et al.* (1972 ▶). For conformations of five-membered rings, see: Schwarz (1973 ▶); Cremer & Pople (1975 ▶); Boeyens & Dobson (1987 ▶). For analysis of absolute structure, see: Flack (1983 ▶), Hooft *et al.* (2008 ▶). For hydrogen bonding in nucleotide chemistry, see: Gilli & Gilli (2009 ▶); Desiraju & Steiner (1999 ▶); Jeffrey (1997 ▶); Nagaswamy *et al.*(2000 ▶) and references therein. For similar UU-4-carbon­yl–imino pairs in RNA structures, see: Ban *et al.* (2000 ▶); Jiang & Patel (1998 ▶).
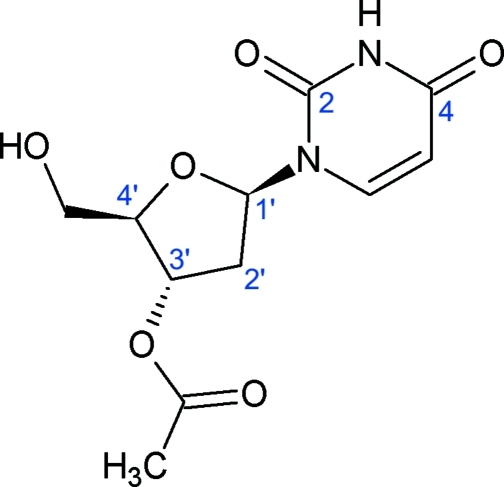


## Experimental

### 

#### Crystal data

C_11_H_14_N_2_O_6_
*M* *_r_* = 270.24
Monoclinic, 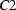

*a* = 22.8919 (4) Å
*b* = 6.8676 (1) Å
*c* = 17.2789 (12) Å
β = 111.307 (8)°
*V* = 2530.8 (2) Å^3^
*Z* = 8
Cu *K*α radiation
μ = 1.00 mm^−1^
*T* = 291 K
0.2 × 0.15 × 0.1 mm

#### Data collection

Rigaku R-AXIS RAPID II imaging plate diffractometer
Absorption correction: multi-scan (*ABSCOR*; Higashi, 1995 ▶) *T*_min_ = 0.84, *T*_max_ = 0.88
11957 measured reflections
4372 independent reflections
2609 reflections with *I* > 2σ(*I*)
*R*_int_ = 0.069

#### Refinement

*R*[*F* ^2^ > 2σ(*F* ^2^)] = 0.048
*wR*(*F* ^2^) = 0.149
*S* = 1.10
4372 reflections
346 parameters
1 restraint
H-atom parameters constrained
Δρ_max_ = 0.24 e Å^−3^
Δρ_min_ = −0.24 e Å^−3^
Absolute structure: Flack (1983 ▶), 1927 Friedel pairs
Flack parameter: 0.0 (2)

### 

Data collection: *CrystalClear-SM Expert* (Rigaku, 2009 ▶); cell refinement: *CrystalClear-SM Expert*; data reduction: *CrystalClear-SM Expert*; program(s) used to solve structure: *SHELXS97* (Sheldrick, 2008 ▶); program(s) used to refine structure: *SHELXL97* (Sheldrick, 2008 ▶); molecular graphics: *ORTEP-3 for Windows* (Farrugia, 1997 ▶) and *Mercury* (Macrae *et al.*, 2008 ▶); software used to prepare material for publication: *PLATON* (Spek, 2009 ▶).

## Supplementary Material

Crystal structure: contains datablocks global, I. DOI: 10.1107/S160053681004938X/zl2331sup1.cif

Structure factors: contains datablocks I. DOI: 10.1107/S160053681004938X/zl2331Isup2.hkl

Additional supplementary materials:  crystallographic information; 3D view; checkCIF report

**Fig. 7.**
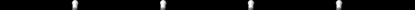
A five-pair fragment of infinitive band of 3'-O-acetyl-2'-deoxyuridine molecules.

Enhanced figure: interactive version of Fig. 7

## Figures and Tables

**Table 1:** Hydrogen-bond geometry (Å, °)

*D*—H⋯*A*	*D*—H	H⋯*A*	*D*⋯*A*	*D*—H⋯*A*
O4—H1⋯O1^i^	0.82	1.98	2.798 (5)	173
N2—H2⋯O22^ii^	0.86	1.98	2.803 (5)	161
N22—H22⋯O2^ii^	0.86	1.99	2.817 (5)	160
O24—H24⋯O21^iii^	0.82	2.02	2.828 (5)	170
C3—H3*A*⋯O22^iv^	0.93	2.24	3.117 (6)	157
C23—H23*A*⋯O2^v^	0.93	2.39	3.254 (5)	154
C24—H24*A*⋯O21^iii^	0.93	2.59	3.381 (5)	143
C31—H31*B*⋯O26^vi^	0.96	2.56	3.428 (8)	150

Symmetry codes: (i) 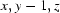; (ii) 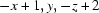; (iii) 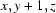; (iv) 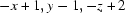; (v) 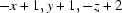; (vi) 
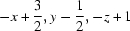
.

**Fig. 1.**
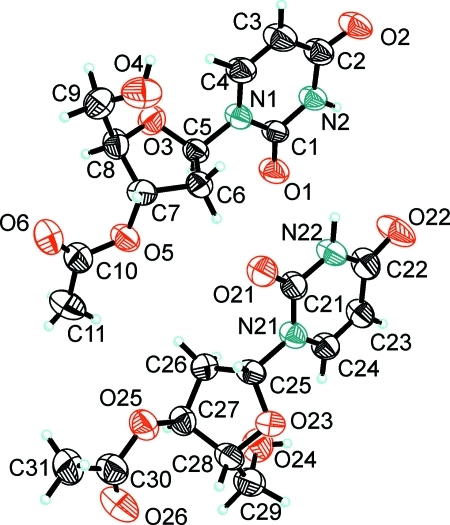
Two molecules of the title compound with displacement ellipsoids drawn at the 50% probability level. H atoms are shown as spheres of arbitrary radius.

**Fig. 2.**
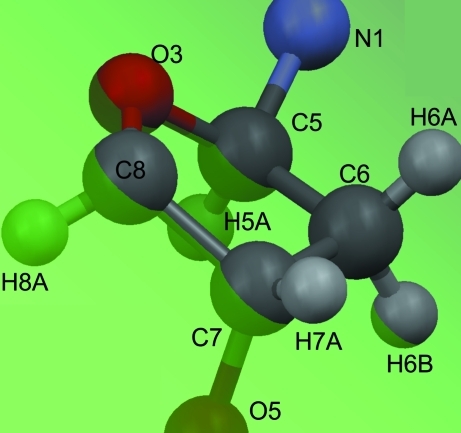
Conformation of deoxyfuranose ring of molecule 1: mean plane through the C5, C7, C8, and O3 atoms.

**Fig. 3.**
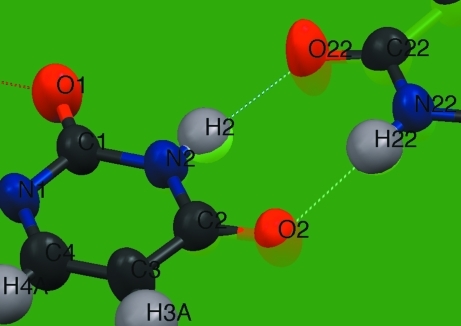
Geometry of resonance-enhanced hydrogen bonding between two uracyl bases.

**Fig. 4.**
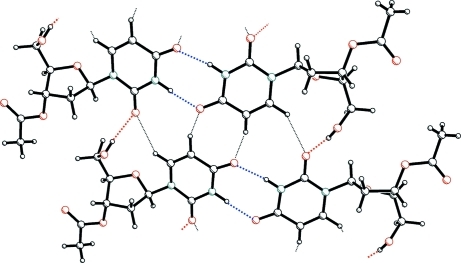
Two pairs of acetyldeoxyuridine molecules connected via N—H···O hydrogen bonds (blue), O—H···O hydrogen bonds (red) and C—H···O hydrogen bonds (black).

**Fig. 5.**
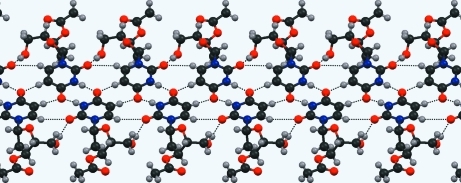
An infinitive chain of acetyldeoxyuridine pairs. View along the a axis.

**Fig. 6.**
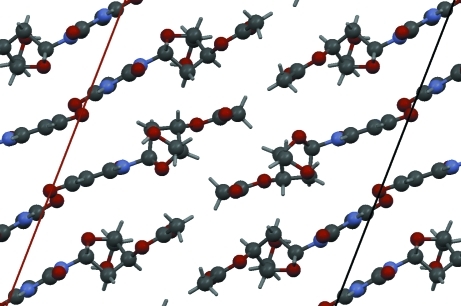
Packing of the title molecules. View along the b axis.

## supplementary crystallographic information

### Comment

Modified nucleosides have received much attention as potential chemotherapeutic agents due to their ability to interfere with the polymerases engaged in replication processes in metastatic or virus invaded cells. In fact, most of the antiviral compounds approved for commercialization are nucleoside analogs which were obtained by modifications of the ribonucleosides or deoxyribonucleosides at a nucleobase moiety, at a carbohydrate moiety or at both of them. The title compound has attracted our attention as a possible intermediate in a synthesis of such agents.

The absolute structure of the title compound is known from the synthetic route which does not affect stereogenic atoms of the starting compound. Nevertheless, we preferred to obtain a direct experimental confirmation using X-ray diffractometry data. Because there are no heavy atoms in a chiral molecule of title compound, Cu *K*α radiation was necessary for determination of the absolute structure.

In the crystal structure of title compound (Fig.1), all bond lengths and bond angles have standard dimensions.

The six-membered rings in both crystallographically independent molecules are flat within 0.01 Å. Figure 2 shows that the furanose ring in the first molecule adopts an envelope conformation with atoms O3, C7, C8, and C5 being within 0.02 Å from their mean plane, and atom C6 at a distance of 0.48 Å. A quantitative analysis of the ring conformations was performed using the method of Cremer and Pople (Cremer & Pople, 1975; Boeyens & Dobson, 1987) for the calculation of parameters of puckering. The polar parameters for the furanose ring are Q = 0.301 (4) and 0.320 (4) Å, Φ = 67.1 (8)° and 66.6 (7) ° for both independent molecules. These suggest the envelope conformation ^2^E (ideal Φ = 72°), slightly distorted towards twist ^2^T_1_ (Φ= 54°), with atoms C(6) and C(26) in corners of the respective envelopes. The conformation of the 3'-substituted 2'-deoxydeoxyuridine reported here is different from the conformation of the unsubstituted 2'-deoxyuridine molecule: in this case Φ = 83 and Φ = 89 ° for two independent molecules which is close to a twisted ^2^T_3_ conformation (Φ=90°) of the furanose ring (CSD code DOURID, Rahman & Wilson, 1972). An ^2^E conformation was observed in several other molecules of the uridine family (see, for example 2'-deoxy-3',5'-diacetyldeoxyuridine (WEVJOX, Luo *et al.*, 2007) Φ = 67 °; 3,5-diacetyluridine (DAURID, de Graaff *et al.*, 1977) Φ =76 °; and 2'-chloro-2'-deoxyuridine (CDURID, Suck *et al.*, 1972) Φ =69 °). ^3^E and ^3^T_2_ conformations exist in uridine (BEURID10, Green *et al.*, 1975) with Φ =282 ° and 273 °. Twisted conformations ^O^T_5 _and ^3^T_4_ are observed in 2'deoxy-2'-fluorodeoxyuridine (BOFWIC, Marck *et al.*, 1982) Φ =339 ° and 2,3,5-triacetyluridine (CIHNIK, Low & Wilson, 1984) Φ =313 °. Therefore, no direct correlation between the substituents, their properties, and the furanose ring conformation is obvious.

In the crystal of the title compound, the two independent acetyldeoxyuridine molecules form a pseudosymmetric dimer of two bases connected *via* two nearly identical N—H···O hydrogen bonds (Table 1, Figure 3). Such pseudosymmetric arrangment corresponds to a UU4^2^ mode of base pairing (Jeffrey, 1997). Relatively short N···O separations (Table 1) demonstrate strong resonance-assisted hydrogen bonds. All eight cycle-forming atoms are located close to the mean plane (Figure 3), making possible π-delocalization of the resonance fragment. This observation is also supported by longer C=O bond lengths in the participating carbonyl groups (1.236 (5) and 1.230 (5) Å) when compared to the other carbonyl groups of the same nucleobase (1.223 (5) and 1.212 (5) Å).

The resulting dimer is further connected with neighboring dimers *via* two similar O—H···O bonds involving the only hydroxy group of deoxyfuranose fragment and the remaining carbonyl oxygen of the base. These interactions result in the formation of infinitive "double bands" along the b axis of the crystal cell (Figure 4). Such a structure can be considered as a primitive self-assembled analogue of an RNA polymer molecule with non-canonical Watson-Crick base pairs. Two examples of similar UU-4-carbonyl-immino pairs in RNA structures can be found in an NMR structure (Jiang & Patel, 1998) and in a low resolution solid state structure (Ban *et al.*, 2000). More information about flipped pyrimidine-pyrimidine mismatches can be found in (Nagaswamy *et al.*, 2000).

The infinitive chains of acetyldeoxyuridine pairs in the title compound are additionally kept together by CH···O interactions involving carbon atoms of the uracyl base and oxygen atoms of carbonyl groups (Table 1, Figure 4 and 5). Similar bonds were observed in various uracyl-containg structures (Desiraju & Steiner, 1999). A short intramolecular contact between carbonyl oxygen O1 and hydrogen atom H5A may additionaly stabilize the conformation of the molecule.

Only weak C—H···O contacts exist between neighboring chains.

### Experimental

The synthesis of the title compound was accomplished *via* a transetherification procedure dubbed "protecting group transfer" (Cabral *et al.*, 2008). 3'-Acetyl-2'-deoxyuridine obtained in this way showed the same properties as the one obtained before by an independent procedure (Smrt & Sorm, 1960). Crystallization from a hexane-acetone system yielded colourless crystals suitable for single-crystal diffractometry (m.p. 460–461 K).

### Refinement

The chirality of the title compound was known from the synthetic route; it was also examined using anomalous scattering. Analysis of the absolute structure using likelihood methods (Hooft *et al.*, 2008) was performed using *PLATON* (Spek, 2009); 1867 Bijvoet pairs were employed. The results confirmed that the absolute structure had been correctly assigned: the probability that the structure is inverted is smaller than 10^-11^ with probability of racemic twinning at 0.001. Because no atom heavier than O is present, the standard deviation of the Flack parameter is relatively high. All H atoms were positioned geometrically with *U*_iso_(H) = 1.2 or 1.5 *U*_eq_(C).

### Crystal data

**Table:**

C_11_H_14_N_2_O_6_	*F*(000) = 1136
*M**_r_* = 270.24	*D*_x_ = 1.418 Mg m^−^^3^
Monoclinic, *C*2	Melting point: 461 K
Hall symbol: C 2y	Cu *K*α radiation, λ = 1.54187 Å
*a* = 22.8919 (4) Å	Cell parameters from 9690 reflections
*b* = 6.8676 (1) Å	θ = 6.8–68.2°
*c* = 17.2789 (12) Å	µ = 1.00 mm^−^^1^
β = 111.307 (8)°	*T* = 291 K
*V* = 2530.8 (2) Å^3^	Block, colourless
*Z* = 8	0.2 × 0.15 × 0.1 mm

### Data collection

**Table:**

Rigaku R-AXIS RAPID II imaging plate diffractometer	4372 independent reflections
Radiation source: fine-focus sealed tube	2609 reflections with *I* > 2σ(*I*)
graphite	*R*_int_ = 0.069
Detector resolution: 10 pixels mm^-1^	θ_max_ = 67.0°, θ_min_ = 6.8°
ω scans	*h* = −27→22
Absorption correction: multi-scan (*ABSCOR*; Higashi, 1995)	*k* = −8→7
*T*_min_ = 0.84, *T*_max_ = 0.88	*l* = −17→20
11957 measured reflections	

### Refinement

**Table:**

Refinement on *F*^2^	Hydrogen site location: inferred from neighbouring sites
Least-squares matrix: full	H-atom parameters constrained
*R*[*F*^2^ > 2σ(*F*^2^)] = 0.048	*w* = 1/[σ^2^(*F*_o_^2^) + (0.0552*P*)^2^ + 0.1596*P*] where *P* = (*F*_o_^2^ + 2*F*_c_^2^)/3
*wR*(*F*^2^) = 0.149	(Δ/σ)_max_ < 0.001
*S* = 1.10	Δρ_max_ = 0.24 e Å^−^^3^
4372 reflections	Δρ_min_ = −0.24 e Å^−^^3^
346 parameters	Extinction correction: *SHELXL97* (Sheldrick, 2008), Fc^*^=kFc[1+0.001xFc^2^λ^3^/sin(2θ)]^-1/4^
1 restraint	Extinction coefficient: 0.0016 (2)
Primary atom site location: structure-invariant direct methods	Absolute structure: Flack (1983), 1927 Friedel pairs
Secondary atom site location: difference Fourier map	Flack parameter: 0.0 (2)

### Special details

**Table:**

Geometry. All e.s.d.'s (except the e.s.d. in the dihedral angle between two l.s. planes) are estimated using the full covariance matrix. The cell e.s.d.'s are taken into account individually in the estimation of e.s.d.'s in distances, angles and torsion angles; correlations between e.s.d.'s in cell parameters are only used when they are defined by crystal symmetry. An approximate (isotropic) treatment of cell e.s.d.'s is used for estimating e.s.d.'s involving l.s. planes.
Refinement. Refinement of *F*^2^ against ALL reflections. The weighted *R*-factor *wR* and goodness of fit *S* are based on *F*^2^, conventional *R*-factors *R* are based on *F*, with *F* set to zero for negative *F*^2^. The threshold expression of *F*^2^ > σ(*F*^2^) is used only for calculating *R*-factors(gt) *etc*. and is not relevant to the choice of reflections for refinement. *R*-factors based on *F*^2^ are statistically about twice as large as those based on *F*, and *R*- factors based on ALL data will be even larger.

### Fractional atomic coordinates and isotropic or equivalent isotropic displacement parameters (Å^2^)

**Table:**

	*x*	*y*	*z*	*U*_iso_*/*U*_eq_	
O1	0.46379 (15)	0.4346 (4)	0.81776 (18)	0.0769 (9)	
O2	0.40640 (14)	0.0919 (4)	1.00331 (19)	0.0794 (9)	
O3	0.46084 (12)	−0.0505 (4)	0.68957 (16)	0.0697 (8)	
O4	0.54767 (15)	−0.3837 (5)	0.7567 (2)	0.0912 (11)	
H1	0.5257	−0.4439	0.7769	0.137*	
O5	0.56917 (13)	0.1405 (4)	0.63582 (18)	0.0689 (8)	
O6	0.59476 (16)	−0.0887 (6)	0.5611 (2)	0.0920 (11)	
N1	0.46541 (16)	0.1042 (5)	0.8110 (2)	0.0590 (9)	
N2	0.43469 (16)	0.2568 (5)	0.9092 (2)	0.0631 (9)	
H2	0.4267	0.3631	0.9297	0.076*	
C1	0.45611 (19)	0.2760 (6)	0.8447 (2)	0.0562 (10)	
C2	0.4246 (2)	0.0868 (7)	0.9442 (3)	0.0698 (12)	
C3	0.4369 (2)	−0.0844 (7)	0.9075 (3)	0.0809 (15)	
H3A	0.4314	−0.2061	0.9274	0.097*	
C4	0.4567 (2)	−0.0696 (7)	0.8439 (3)	0.0743 (13)	
H4A	0.4649	−0.1836	0.8206	0.089*	
C5	0.48799 (18)	0.1107 (6)	0.7418 (2)	0.0563 (10)	
H5A	0.4743	0.2322	0.7107	0.068*	
C6	0.55755 (18)	0.0894 (6)	0.7668 (2)	0.0612 (11)	
H6A	0.5749	0.0100	0.8163	0.073*	
H6B	0.5781	0.2153	0.7767	0.073*	
C7	0.56437 (19)	−0.0114 (6)	0.6914 (2)	0.0609 (11)	
H7A	0.6012	−0.0965	0.7082	0.073*	
C8	0.5048 (2)	−0.1272 (6)	0.6545 (3)	0.0640 (11)	
H8A	0.4877	−0.1063	0.5943	0.077*	
C9	0.5126 (2)	−0.3445 (7)	0.6714 (3)	0.0788 (14)	
H9A	0.4716	−0.4047	0.6559	0.095*	
H9B	0.5339	−0.4016	0.6375	0.095*	
C10	0.5876 (2)	0.0811 (9)	0.5732 (3)	0.0784 (14)	
C11	0.5960 (3)	0.2490 (9)	0.5245 (3)	0.110 (2)	
H11A	0.6366	0.2416	0.5201	0.164*	
H11B	0.5928	0.3680	0.5518	0.164*	
H11C	0.5641	0.2462	0.4699	0.164*	
O21	0.70463 (14)	0.1998 (4)	0.87917 (18)	0.0703 (8)	
O22	0.61778 (17)	0.5604 (4)	1.0291 (2)	0.1020 (13)	
O23	0.78772 (13)	0.6734 (4)	0.82972 (16)	0.0676 (8)	
O24	0.71663 (15)	1.0090 (5)	0.7407 (2)	0.0874 (10)	
H24	0.7181	1.0662	0.7830	0.131*	
O25	0.76788 (13)	0.4633 (5)	0.65709 (18)	0.0737 (9)	
O26	0.80745 (19)	0.6677 (7)	0.5882 (2)	0.1077 (13)	
N21	0.71512 (15)	0.5305 (5)	0.87739 (19)	0.0575 (9)	
N22	0.66228 (16)	0.3867 (5)	0.9545 (2)	0.0647 (9)	
H22	0.6502	0.2828	0.9722	0.078*	
C21	0.6952 (2)	0.3601 (7)	0.9015 (3)	0.0588 (11)	
C22	0.6469 (2)	0.5612 (7)	0.9816 (3)	0.0710 (13)	
C23	0.6674 (2)	0.7311 (6)	0.9510 (2)	0.0638 (12)	
H23A	0.6586	0.8544	0.9663	0.077*	
C24	0.6993 (2)	0.7113 (5)	0.9002 (2)	0.0610 (11)	
H24A	0.7114	0.8228	0.8794	0.073*	
C25	0.74536 (19)	0.5179 (6)	0.8160 (2)	0.0582 (10)	
H25A	0.7679	0.3940	0.8225	0.070*	
C26	0.69949 (18)	0.5388 (7)	0.7270 (2)	0.0625 (11)	
H26A	0.6828	0.4134	0.7034	0.075*	
H26B	0.6651	0.6248	0.7238	0.075*	
C27	0.73937 (19)	0.6245 (6)	0.6843 (2)	0.0614 (11)	
H27A	0.7148	0.7069	0.6375	0.074*	
C28	0.7886 (2)	0.7422 (6)	0.7511 (3)	0.0641 (11)	
H28A	0.8298	0.7140	0.7485	0.077*	
C29	0.7786 (2)	0.9609 (7)	0.7462 (3)	0.0813 (14)	
H29A	0.8087	1.0218	0.7951	0.098*	
H29B	0.7856	1.0113	0.6979	0.098*	
C30	0.7987 (2)	0.5034 (9)	0.6061 (3)	0.0817 (15)	
C31	0.8202 (2)	0.3226 (9)	0.5769 (3)	0.108 (2)	
H31A	0.8428	0.3565	0.5417	0.162*	
H31B	0.7846	0.2443	0.5463	0.162*	
H31C	0.8472	0.2506	0.6240	0.162*	

### Atomic displacement parameters (Å^2^)

**Table:**

	*U*^11^	*U*^22^	*U*^33^	*U*^12^	*U*^13^	*U*^23^
O1	0.111 (2)	0.0498 (18)	0.092 (2)	−0.0016 (18)	0.0627 (19)	0.0023 (17)
O2	0.103 (2)	0.065 (2)	0.098 (2)	0.0078 (17)	0.070 (2)	0.0114 (17)
O3	0.0616 (17)	0.0763 (19)	0.0791 (19)	−0.0050 (16)	0.0350 (15)	−0.0208 (16)
O4	0.087 (2)	0.080 (2)	0.102 (2)	−0.0043 (18)	0.0301 (19)	0.0241 (19)
O5	0.080 (2)	0.0643 (19)	0.0775 (19)	0.0011 (16)	0.0471 (16)	0.0052 (16)
O6	0.108 (3)	0.099 (3)	0.087 (2)	−0.001 (2)	0.058 (2)	−0.018 (2)
N1	0.077 (2)	0.045 (2)	0.072 (2)	0.0068 (17)	0.0471 (19)	0.0022 (17)
N2	0.085 (2)	0.045 (2)	0.078 (2)	0.0013 (18)	0.052 (2)	0.0004 (18)
C1	0.064 (3)	0.048 (2)	0.069 (3)	0.000 (2)	0.039 (2)	0.000 (2)
C2	0.084 (3)	0.060 (3)	0.080 (3)	0.002 (2)	0.048 (3)	0.005 (3)
C3	0.119 (4)	0.049 (3)	0.106 (4)	0.007 (3)	0.078 (3)	0.005 (3)
C4	0.101 (4)	0.047 (3)	0.095 (3)	−0.006 (2)	0.059 (3)	0.001 (2)
C5	0.064 (3)	0.052 (2)	0.065 (2)	−0.004 (2)	0.038 (2)	−0.003 (2)
C6	0.061 (2)	0.065 (3)	0.068 (2)	−0.005 (2)	0.036 (2)	−0.007 (2)
C7	0.061 (3)	0.057 (3)	0.074 (3)	0.001 (2)	0.035 (2)	0.005 (2)
C8	0.078 (3)	0.058 (3)	0.059 (2)	−0.001 (2)	0.029 (2)	−0.005 (2)
C9	0.090 (3)	0.063 (3)	0.089 (3)	−0.006 (3)	0.040 (3)	−0.010 (3)
C10	0.077 (3)	0.097 (4)	0.073 (3)	−0.011 (3)	0.040 (3)	−0.016 (3)
C11	0.135 (5)	0.127 (5)	0.091 (4)	−0.033 (4)	0.069 (4)	0.012 (4)
O21	0.092 (2)	0.0456 (18)	0.086 (2)	0.0012 (16)	0.0480 (17)	−0.0062 (16)
O22	0.160 (3)	0.062 (2)	0.141 (3)	0.013 (2)	0.124 (3)	0.006 (2)
O23	0.0711 (18)	0.0721 (19)	0.0663 (17)	−0.0189 (15)	0.0328 (14)	−0.0018 (15)
O24	0.084 (2)	0.081 (3)	0.103 (3)	0.0105 (18)	0.0398 (18)	−0.015 (2)
O25	0.083 (2)	0.077 (2)	0.078 (2)	−0.0012 (18)	0.0493 (17)	−0.0081 (17)
O26	0.119 (3)	0.125 (3)	0.107 (3)	0.007 (3)	0.074 (2)	0.025 (3)
N21	0.067 (2)	0.052 (2)	0.063 (2)	0.0026 (16)	0.0364 (17)	0.0013 (17)
N22	0.084 (2)	0.050 (2)	0.082 (2)	0.0043 (18)	0.055 (2)	0.0060 (18)
C21	0.071 (3)	0.050 (3)	0.060 (2)	−0.005 (2)	0.030 (2)	0.001 (2)
C22	0.098 (4)	0.049 (3)	0.077 (3)	0.010 (2)	0.045 (3)	0.004 (2)
C23	0.087 (3)	0.046 (3)	0.072 (3)	0.007 (2)	0.045 (3)	−0.001 (2)
C24	0.083 (3)	0.040 (2)	0.069 (3)	0.002 (2)	0.038 (2)	0.005 (2)
C25	0.064 (2)	0.053 (3)	0.068 (3)	−0.001 (2)	0.038 (2)	0.003 (2)
C26	0.061 (3)	0.065 (3)	0.066 (3)	−0.006 (2)	0.029 (2)	−0.007 (2)
C27	0.064 (3)	0.062 (3)	0.064 (2)	0.004 (2)	0.031 (2)	−0.002 (2)
C28	0.066 (3)	0.064 (3)	0.076 (3)	−0.001 (2)	0.043 (2)	0.003 (2)
C29	0.093 (4)	0.071 (3)	0.093 (3)	−0.008 (3)	0.048 (3)	−0.006 (3)
C30	0.068 (3)	0.108 (5)	0.076 (3)	−0.004 (3)	0.034 (3)	−0.002 (3)
C31	0.092 (4)	0.141 (6)	0.113 (4)	−0.020 (4)	0.063 (3)	−0.052 (4)

### Geometric parameters (Å, °)

**Table:**

O1—C1	1.223 (5)	O21—C21	1.212 (5)
O2—C2	1.236 (5)	O22—C22	1.230 (5)
O3—C5	1.421 (5)	O23—C25	1.404 (4)
O3—C8	1.449 (4)	O23—C28	1.445 (4)
O4—C9	1.425 (5)	O24—C29	1.425 (5)
O4—H1	0.8200	O24—H24	0.8200
O5—C10	1.358 (5)	O25—C30	1.342 (5)
O5—C7	1.449 (5)	O25—C27	1.447 (5)
O6—C10	1.207 (6)	O26—C30	1.206 (6)
N1—C4	1.367 (5)	N21—C21	1.375 (5)
N1—C1	1.366 (5)	N21—C24	1.390 (5)
N1—C5	1.467 (4)	N21—C25	1.464 (5)
N2—C2	1.373 (5)	N22—C22	1.379 (5)
N2—C1	1.376 (4)	N22—C21	1.392 (5)
N2—H2	0.8600	N22—H22	0.8600
C2—C3	1.412 (6)	C22—C23	1.429 (6)
C3—C4	1.336 (5)	C23—C24	1.337 (5)
C3—H3A	0.9300	C23—H23A	0.9300
C4—H4A	0.9300	C24—H24A	0.9300
C5—C6	1.498 (5)	C25—C26	1.522 (5)
C5—H5A	0.9800	C25—H25A	0.9800
C6—C7	1.532 (5)	C26—C27	1.488 (5)
C6—H6A	0.9700	C26—H26A	0.9700
C6—H6B	0.9700	C26—H26B	0.9700
C7—C8	1.506 (6)	C27—C28	1.520 (6)
C7—H7A	0.9800	C27—H27A	0.9800
C8—C9	1.519 (6)	C28—C29	1.517 (7)
C8—H8A	0.9800	C28—H28A	0.9800
C9—H9A	0.9700	C29—H29A	0.9700
C9—H9B	0.9700	C29—H29B	0.9700
C10—C11	1.482 (7)	C30—C31	1.489 (7)
C11—H11A	0.9600	C31—H31A	0.9600
C11—H11B	0.9600	C31—H31B	0.9600
C11—H11C	0.9600	C31—H31C	0.9600
			
C5—O3—C8	109.8 (3)	C25—O23—C28	109.6 (3)
C9—O4—H1	109.5	C29—O24—H24	109.5
C10—O5—C7	115.6 (4)	C30—O25—C27	117.6 (4)
C4—N1—C1	120.5 (3)	C21—N21—C24	121.7 (3)
C4—N1—C5	120.9 (4)	C21—N21—C25	117.7 (3)
C1—N1—C5	118.5 (3)	C24—N21—C25	119.9 (3)
C2—N2—C1	127.2 (4)	C22—N22—C21	127.1 (4)
C2—N2—H2	116.4	C22—N22—H22	116.4
C1—N2—H2	116.4	C21—N22—H22	116.4
O1—C1—N1	122.7 (4)	O21—C21—N21	124.0 (4)
O1—C1—N2	122.5 (4)	O21—C21—N22	122.0 (4)
N1—C1—N2	114.7 (4)	N21—C21—N22	114.0 (4)
O2—C2—N2	120.1 (4)	O22—C22—N22	119.3 (4)
O2—C2—C3	125.3 (4)	O22—C22—C23	125.5 (4)
N2—C2—C3	114.6 (4)	N22—C22—C23	115.2 (4)
C4—C3—C2	119.3 (4)	C24—C23—C22	119.4 (4)
C4—C3—H3A	120.4	C24—C23—H23A	120.3
C2—C3—H3A	120.4	C22—C23—H23A	120.3
C3—C4—N1	123.5 (4)	C23—C24—N21	122.5 (4)
C3—C4—H4A	118.2	C23—C24—H24A	118.7
N1—C4—H4A	118.2	N21—C24—H24A	118.7
O3—C5—N1	107.0 (3)	O23—C25—N21	108.2 (3)
O3—C5—C6	106.3 (3)	O23—C25—C26	106.2 (3)
N1—C5—C6	114.5 (3)	N21—C25—C26	113.1 (3)
O3—C5—H5A	109.6	O23—C25—H25A	109.7
N1—C5—H5A	109.6	N21—C25—H25A	109.7
C6—C5—H5A	109.6	C26—C25—H25A	109.7
C5—C6—C7	103.0 (3)	C27—C26—C25	102.5 (3)
C5—C6—H6A	111.2	C27—C26—H26A	111.3
C7—C6—H6A	111.2	C25—C26—H26A	111.3
C5—C6—H6B	111.2	C27—C26—H26B	111.3
C7—C6—H6B	111.2	C25—C26—H26B	111.3
H6A—C6—H6B	109.1	H26A—C26—H26B	109.2
O5—C7—C8	112.0 (3)	O25—C27—C26	106.8 (3)
O5—C7—C6	107.1 (3)	O25—C27—C28	110.8 (3)
C8—C7—C6	104.0 (3)	C26—C27—C28	104.6 (3)
O5—C7—H7A	111.2	O25—C27—H27A	111.4
C8—C7—H7A	111.2	C26—C27—H27A	111.4
C6—C7—H7A	111.2	C28—C27—H27A	111.4
O3—C8—C7	106.9 (3)	O23—C28—C29	108.8 (4)
O3—C8—C9	109.1 (4)	O23—C28—C27	106.3 (3)
C7—C8—C9	114.3 (4)	C29—C28—C27	115.4 (4)
O3—C8—H8A	108.8	O23—C28—H28A	108.7
C7—C8—H8A	108.8	C29—C28—H28A	108.7
C9—C8—H8A	108.8	C27—C28—H28A	108.7
O4—C9—C8	111.6 (4)	O24—C29—C28	111.1 (4)
O4—C9—H9A	109.3	O24—C29—H29A	109.4
C8—C9—H9A	109.3	C28—C29—H29A	109.4
O4—C9—H9B	109.3	O24—C29—H29B	109.4
C8—C9—H9B	109.3	C28—C29—H29B	109.4
H9A—C9—H9B	108.0	H29A—C29—H29B	108.0
O6—C10—O5	122.0 (5)	O26—C30—O25	122.4 (5)
O6—C10—C11	126.8 (5)	O26—C30—C31	125.9 (5)
O5—C10—C11	111.2 (5)	O25—C30—C31	111.6 (5)
C10—C11—H11A	109.5	C30—C31—H31A	109.5
C10—C11—H11B	109.5	C30—C31—H31B	109.5
H11A—C11—H11B	109.5	H31A—C31—H31B	109.5
C10—C11—H11C	109.5	C30—C31—H31C	109.5
H11A—C11—H11C	109.5	H31A—C31—H31C	109.5
H11B—C11—H11C	109.5	H31B—C31—H31C	109.5
			
C4—N1—C1—O1	179.5 (4)	C24—N21—C21—O21	175.5 (4)
C5—N1—C1—O1	2.5 (6)	C25—N21—C21—O21	4.8 (6)
C4—N1—C1—N2	−3.3 (6)	C24—N21—C21—N22	−4.2 (6)
C5—N1—C1—N2	179.8 (3)	C25—N21—C21—N22	−174.8 (3)
C2—N2—C1—O1	−179.8 (4)	C22—N22—C21—O21	−177.5 (4)
C2—N2—C1—N1	3.0 (6)	C22—N22—C21—N21	2.1 (6)
C1—N2—C2—O2	178.5 (4)	C21—N22—C22—O22	−179.5 (4)
C1—N2—C2—C3	−1.3 (7)	C21—N22—C22—C23	0.1 (7)
O2—C2—C3—C4	−179.8 (5)	O22—C22—C23—C24	179.3 (4)
N2—C2—C3—C4	−0.1 (7)	N22—C22—C23—C24	−0.3 (6)
C2—C3—C4—N1	−0.5 (8)	C22—C23—C24—N21	−1.8 (7)
C1—N1—C4—C3	2.3 (7)	C21—N21—C24—C23	4.3 (6)
C5—N1—C4—C3	179.2 (4)	C25—N21—C24—C23	174.7 (4)
C8—O3—C5—N1	−144.8 (3)	C28—O23—C25—N21	−144.4 (3)
C8—O3—C5—C6	−22.1 (4)	C28—O23—C25—C26	−22.7 (4)
C4—N1—C5—O3	36.1 (5)	C21—N21—C25—O23	−151.1 (3)
C1—N1—C5—O3	−146.9 (3)	C24—N21—C25—O23	38.1 (5)
C4—N1—C5—C6	−81.4 (5)	C21—N21—C25—C26	91.5 (4)
C1—N1—C5—C6	95.6 (5)	C24—N21—C25—C26	−79.3 (5)
O3—C5—C6—C7	31.3 (4)	O23—C25—C26—C27	32.7 (4)
N1—C5—C6—C7	149.2 (3)	N21—C25—C26—C27	151.3 (3)
C10—O5—C7—C8	−78.8 (5)	C30—O25—C27—C26	170.3 (4)
C10—O5—C7—C6	167.8 (3)	C30—O25—C27—C28	−76.3 (5)
C5—C6—C7—O5	90.1 (4)	C25—C26—C27—O25	88.0 (4)
C5—C6—C7—C8	−28.7 (4)	C25—C26—C27—C28	−29.6 (4)
C5—O3—C8—C7	3.2 (4)	C25—O23—C28—C29	128.5 (4)
C5—O3—C8—C9	127.3 (4)	C25—O23—C28—C27	3.6 (4)
O5—C7—C8—O3	−99.0 (4)	O25—C27—C28—O23	−97.5 (4)
C6—C7—C8—O3	16.3 (4)	C26—C27—C28—O23	17.3 (4)
O5—C7—C8—C9	140.2 (4)	O25—C27—C28—C29	141.8 (4)
C6—C7—C8—C9	−104.5 (4)	C26—C27—C28—C29	−103.5 (4)
O3—C8—C9—O4	−72.2 (5)	O23—C28—C29—O24	−68.0 (5)
C7—C8—C9—O4	47.4 (6)	C27—C28—C29—O24	51.4 (5)
C7—O5—C10—O6	5.4 (7)	C27—O25—C30—O26	6.2 (7)
C7—O5—C10—C11	−175.3 (4)	C27—O25—C30—C31	−174.6 (4)

### Hydrogen-bond geometry (Å, °)

**Table:**

*D*—H···*A*	*D*—H	H···*A*	*D*···*A*	*D*—H···*A*
O4—H1···O1^i^	0.82	1.98	2.798 (5)	173
N2—H2···O22^ii^	0.86	1.98	2.803 (5)	161
N22—H22···O2^ii^	0.86	1.99	2.817 (5)	160
O24—H24···O21^iii^	0.82	2.02	2.828 (5)	170
C3—H3A···O22^iv^	0.93	2.24	3.117 (6)	157
C23—H23A···O2^v^	0.93	2.39	3.254 (5)	154
C24—H24A···O21^iii^	0.93	2.59	3.381 (5)	143
C31—H31B···O26^vi^	0.96	2.56	3.428 (8)	150

Symmetry codes: (i) *x*, *y*−1, *z*; (ii) −*x*+1, *y*, −*z*+2; (iii) *x*, *y*+1, *z*; (iv) −*x*+1, *y*−1, −*z*+2; (v) −*x*+1, *y*+1, −*z*+2; (vi) −*x*+3/2, *y*−1/2, −*z*+1.
